# Application of the Swimming Pool Backwash Water Recovery System with the Use of Filter Tubes

**DOI:** 10.3390/molecules26216620

**Published:** 2021-10-31

**Authors:** Waldemar Studziński, Wojciech Poćwiardowski, Weronika Osińska

**Affiliations:** 1 Faculty of Chemical Technology and Engineering, Bydgoszcz University of Science and Technology, Seminaryjna 3, 85-326 Bydgoszcz, Poland; Wojciech.Pocwiardowski@pbs.edu.pl; 2 Research and Development Center AS PRODUKT, Zajezierze 5c, 88-140 Gniewkowo, Poland; w.osinska@asprodukt.com

**Keywords:** backwash water, swimming pool, management of backwash water, ultrafiltration

## Abstract

During the operation of swimming pools, large losses of water from the backwash of swimming pool filters are observed. This water is often discharged into sewers or used to sprinkle sports grounds. The aim of the research was to design and build an installation for purification and recovery of backwash water (BWW). It consists of flocculation, pre-filtration, and ultrafiltration based on filter tubes and ozone disinfection. Backwash water treatment installation contributes to purification and improvement of water quality. The effectiveness of the removal of microbial contamination with the use of the system was over 99%. The high efficiency of removing physicochemical impurities was also achieved. Water turbidity was reduced from 96.9 NTU to 0.13 NTU. After using the system, the oxidability of water decreased from 6.26 mg O_2_∙dm^−3^ to 0.4 mg O_2_∙dm^−3^. When using the system, a reduction of total organic carbon by 80% was also noticed. After the treatment process, water meets the strict criteria and can be returned to the pool system of water as fresh water with parameters of supply water—directly to the overflow tank. It has been shown that up to 96% of water can be recovered with the technology. The cost comparison showed annual savings of over EUR 9000.

## 1. Introduction

Taking into account the periodic water deficits and growing prices of water and sewage disposal, it is reasonable to use solutions enabling rational water management and reducing the costs of operating swimming pool facilities [1].

Therefore, pool water installations, more and more often, work in a closed circuit system, enabling the extension of the time of complete water change in the swimming pool basin for up to 1 year [2]. The purpose of the hydraulic system in swimming pools is to maintain the water circulation in the following system: swimming pool—treatment station—disinfection—swimming pool. To ensure that the pool water meets parameters contained in the applicable standards, it is necessary to partially replace it with fresh tap water, appropriate mixed water treatment and ensure optimal flow through the pool basin [3,4,5,6].

When analyzing the possibilities of reducing water consumption and wastewater discharge in swimming pools, more and more attention is paid to filtration washings (backwash water—BWW) [7,8]. The need for regular backwashing of filter beds in the technological systems of swimming pool water generates huge, unproductive losses of water [9]. The filter beds are rinsed once every 2–4 days to meet the physicochemical and sanitary requirements of water quality [10,11,12,13].

It is estimated that in Poland over 2,800,000 m^3^/year of backwash water is lost, which causes costs of over EUR 4 million as a result of discharging backwash to the sewage system [9]. Moreover, water used to backwash the filter beds is usually taken from the technological system in which it was previously heated. For this reason, its discharge into the sewage system is also a waste of energy used to heat it [8,14].

Among the many possibilities of using water discharged from swimming pool facilities, apart from discharges to the sewage system, the most common are sprinkling sports areas (tennis courts, football fields), powering the toilet flushing system and irrigating various salinity-tolerant plants (halophytes). In practice, when planning a direct discharge of BWW to the environment, particular attention should be paid to the content of pollutants in them. The studies conducted so far show that it is not possible to use backwash water from filter beds without its treatment, due to the high content of total suspended solids and residual chlorine [14].

In order to reduce backwash water losses, water treatment systems are sought that will ensure water free from pathogenic microorganisms (bacteria, viruses, fungi, staphylococci, cysts), clear, characterized by satisfactory aesthetic quality, not irritating eyes, the nose and other mucous membranes, and without organic contamination.

So far, the possibilities of backwash water management have been tested using simple but relatively expensive individual processes (adsorption, coagulation, filtration, electrocoagulation, electroflocculation) and devices (for example, sedimentation tanks, settling tanks, clarifiers or settling tanks coupled with a coagulant chamber). However, this does not purify the water sufficiently and does not meet stringent standards [8,9,15,16,17,18,19].

Combined systems using membrane techniques (ultrafiltration UF and microfiltration MF) are an interesting alternative enabling the rational management of water and wastewater in swimming pool systems [1,18,20,21,22]. In a few cases, by using a membrane to reuse BWW, a significant reduction in the operating costs of swimming pools can be achieved [3]. Various pilot and full-scale water treatment plants are being designed which consist of processes such as sand filtration or activated carbon filtration combined with UF and with oxidation processes. The indicated installations can also be used in water reuse systems in swimming pools [6]. Such systems can recover more than 90% of the water. However, there is a constant search for new, more effective systems for the recovery of swimming pool water.

Therefore, the aim of the studies was to design and apply an innovative backwash water treatment system based on flocculation, pre-filtration, ultrafiltration with the use of filter tubes and ozonation, which in future may be used in swimming pool circuits. A novelty is the use of recycled tubes after hemodialysis in the system, which have not been used in swimming pool systems so far. The effectiveness of the system in removing microbiological and physicochemical contamination was tested in this work. 

## 2. Results and Discussion

**Figure 1:**
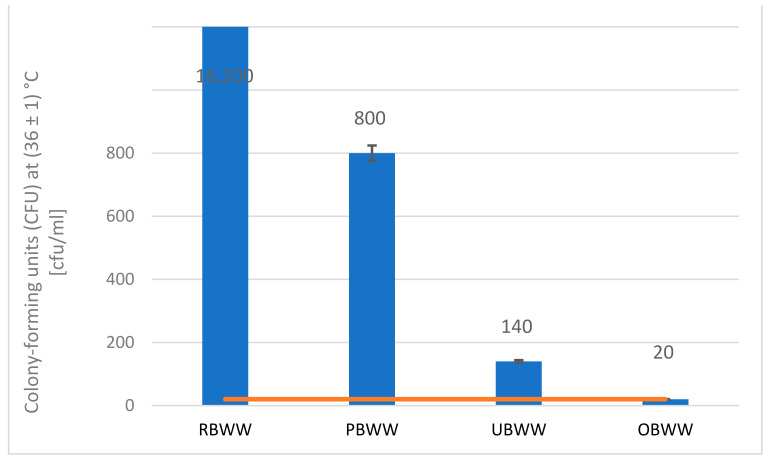
Change in CFU at (36 ± 1) °C during the treatment process: raw backwash water (RBWW), pre-filter backwash water (PBWW), ultrafiltration backwash water (UBWW), ozonation backwash water (OBWW).

In the first step, the effectiveness of the backwash water treatment system was tested in terms of removing microbiological contamination. Based on the results, it was found that the sampled backwash water was not contaminated with Escherichia coli and coagulase-positive staphylococci (Table S1). However, colony-forming units (CFU) at (36 ± 1) °C in the backwash water were very high, amounting to 16,200 cfu mL^−1^. The tests performed indicate a significant reduction of microorganisms during filtration (pre-filter and ultrafiltration). After the ultrafiltration process, CFU at (36 ± 1) °C was reduced by over 99%. However, only the ozonation of water achieved a parameter value that met the requirements for water introduced into the swimming pool basin from the circulation system (Figure 1). The ozonation process is also an important element that protects against recontamination of water. 

The bacterium Pseudomonas aeruginosa turned out to be the most problematic. The results of the test indicate that these bacteria are common in backwash water. Additionally, Pseudomonas aeruginosa is resistant to many disinfectants [23]. 

**Figure 2:**
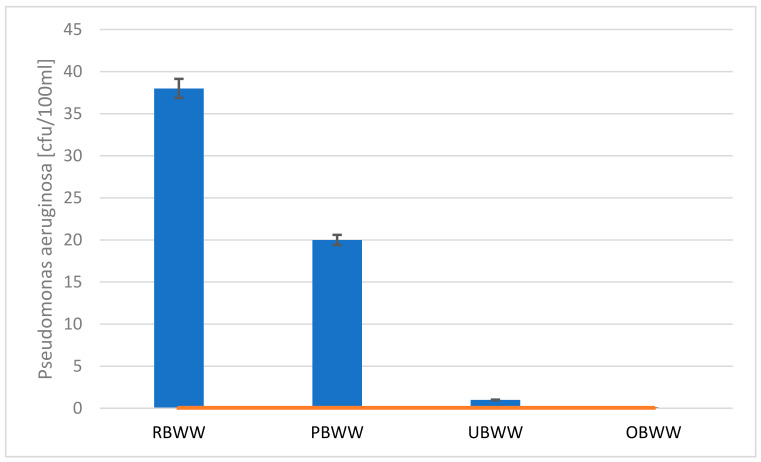
Change in the number of *Pseudomonas aeruginosa* bacteria during treatment process: raw backwash water (RBWW), pre-filter backwash water (PBWW), ultrafiltration backwash water (UBWW), ozonation backwash water (OBWW).

Pre-filter reduced Pseudomonas aeruginosa by 48%, the next process (ultrafiltration) caused a loss of bacteria in water by 98%. In the final stage of the process, water was ozonated. It resulted in complete removal of Pseudomonas aeruginosa bacteria from the system (Figure 2). Finally, water meets the requirements for supplying the swimming pool basin (0 cfu∙mL^−1^).

The sand filter is an effective step in water purification from microbial contamination. When water flows downward through the filter bed, it enters the intensely active biofilm layer, where various microorganisms entrap, digest and break down the organic matter contained within it [24,25]. Yusuf, using a slow sand filter, reduced *E. coli* present in water by 69% [26]. On the other hand, Bolster removed 23–42% *E. coli*, Enterococci, and Salmonella when using a sand filter but when he used an activated carbon-coated sand filter, the efficiency increased to 48–80% [27]. Bagundol et al. [28] reported that the effectiveness of the removal of microorganisms through a sand filter depended on the depth filter material, biofilm layers and flow rate water through the filter, while concluding that the sand filter will not remove completely microorganisms. On the other side, Podaru et al. [29] using the microfiltration-ultrafiltration pilot plant, which consisted of a microfilter and ultrafiltration membranes, achieving the reduction of *E. coli* bacteria in the range of 35.79 and 95.03%. The final results we have obtained are better than those indicated.

Although the concentration of CFU was drastically decreased and met the stringent requirements in our study, it is worth remembering that various microorganisms can go into a dormant state under the influence of environmental stress, in which cells are viable but not culturable (VBNC) [30]. The VBNC state is a bet-hedging strategy for long-term survival under stressful conditions. Some microorganisms can be resuscitated under favorable environmental conditions, for example a yeast of the *Candida* sp. [31]. It is important to ensure effective disinfection to keep the water safe and to prevent re-contamination [32,33]. Often, for reasons of low cost, chlorination is carried out. However, ozone treatment due to its high oxidizing potential and bactericidal properties achieves a higher level of disinfection than chlorine [28,34]. The effectiveness and necessity of ozonation in swimming pool systems is confirmed by the research conducted by Wyczarska-Kokot [33]. Ozonation was effective in reducing *P. aeruginosa*, *E. coli*, *Legionella* sp. and the total number of bacteria [33]. In our case, disinfection is carried out in the washing cleaning process, and then in the swimming pool.

In the next step of studies, it was checked how the backwash water treatment installation changes the selected physicochemical parameters.

**Figure 3:**
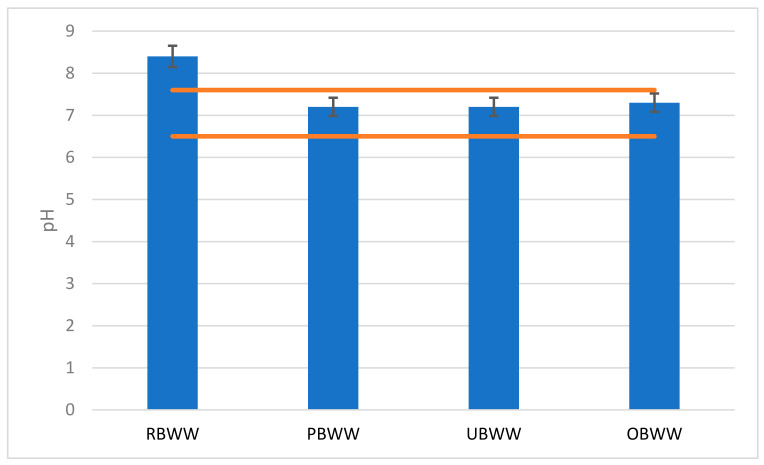
Change of backwash water pH during the treatment process: raw backwash water (RBWW), pre-filter backwash water (PBWW), ultrafiltration backwash water (UBWW), ozonation backwash water (OBWW).

In the samples tested, the backwash water pH values ranged from pH = 8.4 (RBWW) to pH = 7.2 (PBWW and UBWW) (Figure 3). Only the sample of raw backwash water showed a pH slightly above the value specified for the swimming pool water, i.e., pH = 6.5–7.6.

Maintaining the correct pH level of the pool water is very important as it affects the effectiveness of treatment. During disinfection with chlorine compounds, keeping the value in the range of pH 7.2–7.6 will make it fully effective.

Ozone was chosen due to its strong disinfecting and oxidizing properties in swimming pool water treatment systems. In the analyzed backwash water samples, the highest ozone concentration of 0.03 mg O_3_∙dm^−3^ was determined in the backwash water sample after ozonation. In other samples, the ozone concentration was determined in trace amounts i.e., 0.01. In none of the samples the ozone content exceeded the limit value for swimming pool water, i.e., 0.05 mg O_3_∙dm^−3^.

Ozone was also chosen in order to reduce the amount of chlorinated disinfection by-products formed. It is known that chlorine solution poured into water decomposes into, among others, hypochlorous acid (HOCl) and hypochlorite ion (OCl−). When the chlorine compounds finish oxidizing the bacteria, they combine with another chemical. This process leads to the formation of disinfection by-products (DBPs) [35,36,37].

It was found that the tested backwash water was not contaminated with trihalomethanes (significantly below the standard for swimming pool water). In the analyzed samples of backwash water, the total THM did not exceed the limit value for swimming pool water, i.e., 0.1 mg∙dm^−3^ and ranged from 0.0261 mg∙dm^−3^ (RBWW) to 0.011 mg∙dm^−3^ (OBWW). In the analyzed samples of backwash water, the content of free chlorine did not exceed 0.05 mg Cl_2_∙dm^−3^ and bounded chlorine 0.08 mg Cl_2_∙dm^−3^, respectively.

**Figure 4:**
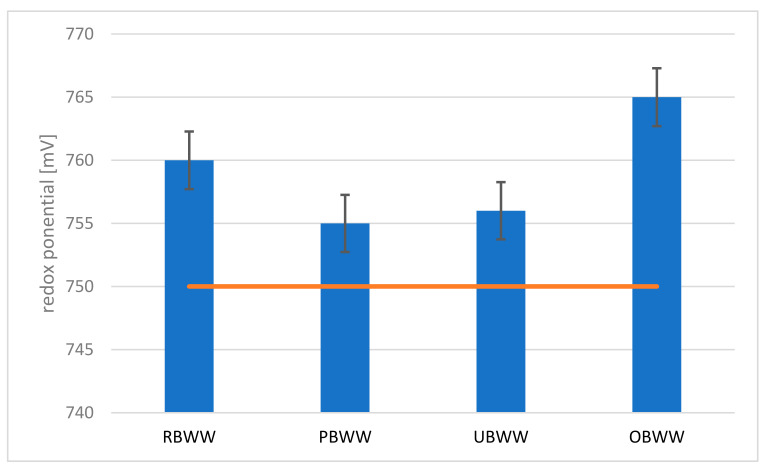
Changes in redox potential at the individual treatment stages: raw backwash water (RBWW), pre-filter backwash water (PBWW), ultrafiltration backwash water (UBWW), ozonation backwash water (OBWW).

In the tested backwash water samples, the redox potential was 760 mV in RBWW, while in PBWW it was 765 mV (Figure 4). Based on the results, it can be concluded that it is possible to use the water recovered from backwash water to supply the swimming pool basin. The pool water should have a redox potential > 750 mV. Such water parameters inform good reduction and oxidation capacity and the destruction of microorganisms in the disinfection process, and as a result, protect bathers against the risk of infection during bathing.

Studies have shown that the system has little effect on the redox potential of the water.

**Figure 5:**
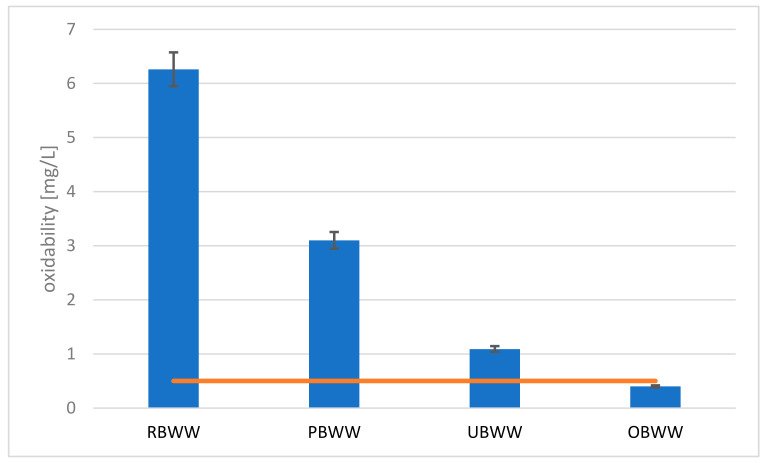
Changes in oxidability at individual stages of treatment: raw backwash water (RBWW), pre-filter backwash water (PBWW), ultrafiltration backwash water (UBWW), ozonation backwash water (OBWW).

Oxidability is another important parameter for swimming pool water, because its high value is associated with the presence of organic substances (including microbial contamination) and easily oxidability inorganic substances in water. The backwash water is usually characterized by high oxidability and the greatest decrease occurs after pre-filtration because the sand filter well cleans water from organic suspensions. In the tested backwash water samples, the raw backwash water was characterized by the highest oxidability equal to 6.26 mg O_2_∙dm^−3^. In the other samples, it was 3.1 mg O_2_∙dm^−3^ (RBWW), 1.09 mg O_2_∙dm^−3^ (UBWW) and 0.4 mg O2∙dm^−3^ (OBWW), respectively. At the final stage, water meets the criteria of the DIN standard for swimming pool water (Figure 5).

Moreover, the oxidability of swimming pool water should not be higher than 4.0 mg O_2_ ∙ dm^−3^ (as the difference between the oxidability of water from the swimming pool basin and the oxidability of water replenishing the swimming pool circuit, usually tap water).

**Figure 6:**
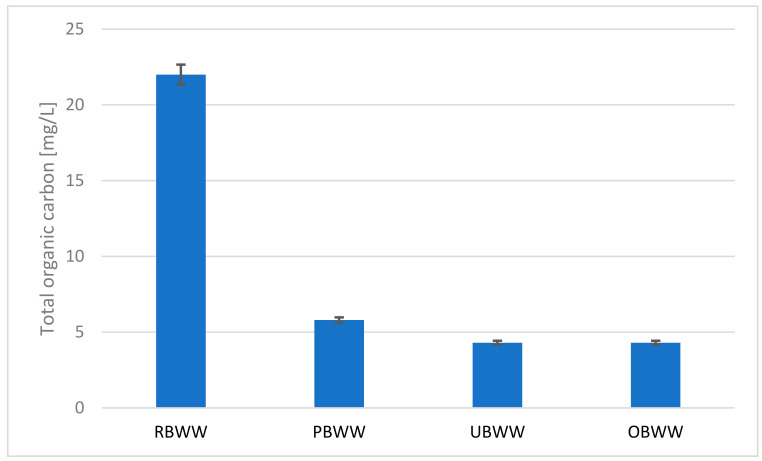
Changes in the TOC content during the treatment process: raw backwash water (RBWW), pre-filter backwash water (PBWW), ultrafiltration backwash water (UBWW), ozonation backwash water (OBWW).

High content of the oxidability can be explained by impurities of organic origin (carbon compounds). It is confirmed by the high value of TOC in raw backwash water (Figure 6). The system allowed for a good reduction of this parameter.

The filtration process removes solid particles very well, but organic compounds in water are less effective [1]. Christensen, who investigated the classic sand filter and the coated filter for treated pool water, achieved TOC reductions of up to 9% [38]. Reibmann reduced dissolved organic carbon (DOC) [1] by 11% with UF. Corina-Petronela et al. [39] using nanofiltration, achieved a chlorophenols removal efficiency of up to 85%. This result is similar to what we achieved after using a sand filter and ultrafiltration, as the TOC was reduced by 80%. The effectiveness of water purification from organic compounds by filtration depends on operational parameters such as: pressure, operating mode, membrane cleaning and the initial concentration of organic compounds [39]. Ozonation, despite the fact that it is an effective method of oxidation of organic compounds in water [40], in this case did not reduce the TOC value after ultrafiltration.

**Figure 7:**
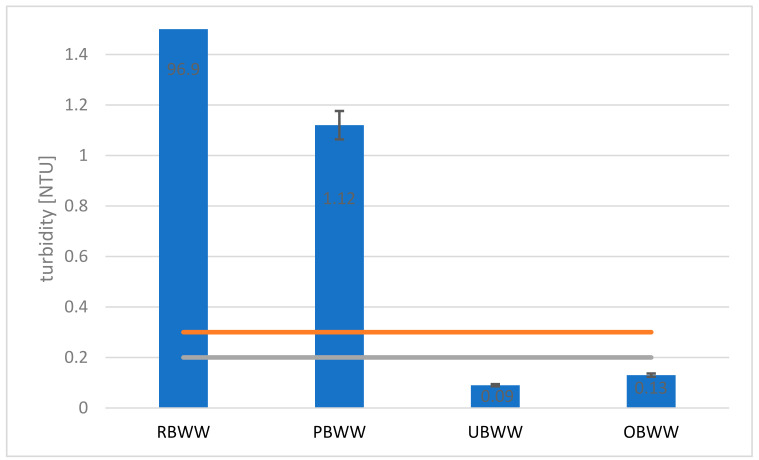
Changes in the turbidity values of backwash water after each process: raw backwash water (RBWW), pre-filter backwash water (PBWW), ultrafiltration backwash water (UBWW), ozonation backwash water (OBWW).

An important parameter in terms of the utility and aesthetic value of swimming pool water is turbidity. Increased water turbidity is an indicator of poor water quality and a higher probability of pathogenic microorganisms growth. In the analyzed samples, the turbidity value of the raw backwash water (RBWW) sample was 96.9 NTU and the preliminary filter backwash (PBWW) samples were 1.12 NTU, which indicates a very good action of the pre-filter (Figure 7). The result is similar to that received by other scientists. Yusuf, after using a sand filter with activated carbon and gravel, observed a turbidity decrease by 86% [26]. Yari [41] using a quick sand filter assisted by electrocoagulation, decreased turbidity in backwash water from 83 to over 90%.

However, the use of only one method of backwash water treatment is not enough to use the filtrate to supply the swimming pool basin. The turbidity values of backwash water after ultrafiltration (UBWW) and ozonation (OBWW) were 0.09 NTU and 0.13 NTU, respectively, and thus did not exceed the permissible value, i.e., 0.2 NTU [42] and 0.3 NTU [43], required for water supplied to the swimming pool basin. Ultimately, using the system, the turbidity was reduced by 99.9%. The result is better than that of Podaru [29] who used the microfiltration-ultrafiltration pilot plant; they reduced the turbidity in the range of 57–98%.

### Cost Efficiency

The biggest difference, and also a novelty, is the use of recycled tubes after hemodialysis in the system, which have not been used in swimming pool systems so far. The commercial systems use ultrafiltration membranes and/or reverse osmosis. The system uses a simple sand filter, not a combined sand filter with activated carbon. It also replaces common disinfection with chlorine compounds. Comparing the system with filter tubes with other systems for water treatment, it can be stated that the presented system is 2–7 times cheaper than the commercial systems available on the market. The system presented in the article also provides one of the largest water recoveries (clean drinking water). The comparison of selected parameters with commercial systems is presented in Table S2.

By focusing on the costs of backwash water treatment with the use of a system with filter tubes, you can reduce the total cost of operating the swimming pool installation and the costs associated with pool water supply. In addition, the energy consumption for water heating is much lower when using the presented system. Table 1 compares the projected pool costs with and without backwash water recovery. The calculations were made for a recreational pool with a total volume of 189 m^3^.

**Figure 8:**
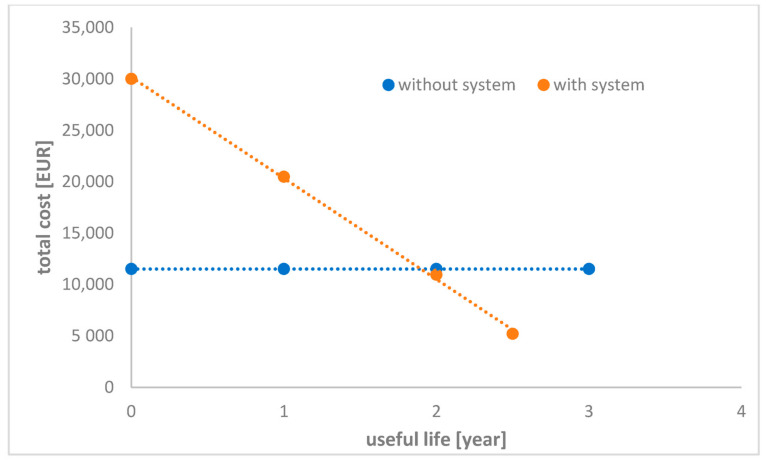
Comparison of costs with and without a backwash water recovery system.

As can be seen, about 3830 m^3^ of water can be reused every year. Based on average wastewater and drinking water costs, the cost reduction using a filter tube system can be around ~7660 EUR/year. Additionally, the annual savings in energy consumption have been calculated to be around ~EUR 3158. The investment cost for the installation with filter tubes has been calculated at EUR 30,000. Operating costs such as service costs, backwash costs, energy costs (pumps), system disinfection, supervision and inspection costs calculated for the system are at EUR 1290/year. Based on these calculations, the installation costs can be compared without and with a system with filter tubes. Figure 8 shows the annual costs depending on the useful life for both.

As is evident after a service life of more than 2 years, the costs of the filter tube system are below operating costs without a backwash water recovery system.

## 3. Materials and Methods

### 3.1. Characteristics of Backwash Water

The studies were performed using raw backwash water from pressure filters (gravel-coal bed), which are part of the hydraulic system in an indoor public swimming pool. The pool uses a vertical water flow system. Water shortages resulting from evaporation, splashing and the need to rinse the filter beds are replenished with water from the water supply network to the retention reservoir. In the swimming pool technology, sodium chlorate (I) is used for disinfection. The swimming pool had a constant heavy load of 66 people per hour. Detailed data on the swimming pool facility are included in the supporting materials Table S3.

Samples for microbiological and physicochemical tests were collected once a week for 5 weeks. Backwash water samples were collected after the swimming pool was closed and after rinsing the filter beds. The values of the tested physicochemical and microbiological parameters of raw backwash water collected at 5 different dates differed from each other by no more than 5% of the values of the parameters tested.

**Figure 9:**
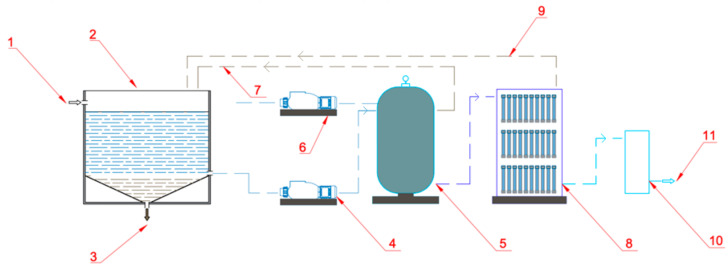
Installation for backwash water purification: 1—raw backwash water; 2—wash water reservoir—flocculation stage; 3—outflow to the sewage system; 4—circulation pump; 5—pre-filter filled with sand; 6—pre-filter pump; 7—backwash water from the pre-filter; 8—ultrafiltration system; 9-backwash water from ultrafiltration system; 10—tank with ozonation; 11—clean water for the swimming pool circuit.

In the study, an innovative, full-size closed-loop backwash water purification installation was designed and applied. The system consists of flocculation, pre-filter, ultrafiltration and ozonation (Figure 9).

First, raw backwash water is directed to the wash water reservoir, where the flocculation stage takes place. The flocculation process is carried out with the use of anionic flocculant at a concentration of 3 g of granules per 1 m^3^ of water. Water from above the dispersed fraction is sucked in by a circulating pump equipped with a pre-filter and is pumped onto a pre-filter filled with sand. Mechanical impurities, suspended solids and colloidal particles are removed on the pre-filter. A sand filter with a diameter of ø 800 mm was used. The filtration speed was 7.5–10 m∙h^−1^ and washing 50 m∙h^−1^, respectively. The filter bed in the filter was rinsed in the counter-current with water taken from the overflow tank of the swimming pool circuit. Backwash water from the installation was re-circulated to the backwash water tank. The sand filter was connected to the ultrafiltration system.

The ultrafiltration system was built from 60 recycled tubes after hemodialysis, replacing the filtration membranes used so far. After medical use, the filters are cleaned, sterilized and assembled to purify water under pressure. The filter horns consist of thousands of fibers (Figure S1), and each fiber acts as a nephron, enabling removal of up to 99.9% of bacteria, viruses, organic substances, opacifying, suspended and colloidal agents in feed water. The details of the tube are presented in Table 2.

Additionally, the use of tubes ensures minimal loss of tap water from 0.1% to 0.8% in this stage. This technology is an incremental innovation on a global scale. The filter tubes were rinsed with tap water and returned to the backwash water tank.

In the last stage of the process, water was disinfected with ozone (ozonator efficiency up to 5 g∙h^−1^), and then discharged to the overflow tank of the swimming pool circuit. The efficiency of the backwash water treatment installation is 5.5 m^3^∙h^−1^. The use of the system allows the recovery of 96% of the water. Additionally, by using the system, we save energy. The water returned to the pool is at a temperature similar to the water in the system (loss only 1 °C), so we do not waste any energy that would otherwise be spent on the pre-heating of incoming water from waterworks.

### 3.2. Microbiological and Physicochemical Tests of Raw and Treated Backwash Water

Microbiological and physicochemical tests were carried out on raw and purified backwash water from the backwash water treatment installation. The study on the degree of contamination of backwash water took into account the basic comparative parameters for swimming pool water, in accordance with the guidelines of the applicable Polish regulation of the Minister of Health of 9 November 2015 on the requirements to be met by water in swimming pools [43] and the German standard DIN 19643-1: 2012-11 [42] (Tables S4 and S5).

The following microbiological parameters were tested: *Escherichia coli*; *Pseudomonas aeruginosa*; coagulase positive *staphylococci*; *Legionella* sp.; colony-forming units (CFU) at (36 ± 1) °C.

The following physicochemical parameters were tested: ammonium ion; nitrates; oxidizability; trichloromethane (chloroform); ΣTHM (chloroform, bromodichloromethane, dibromochloromethane, bromoform); free chlorine; combined chlorine; ozone; turbidity; pH; redox potential; temperature; total organic carbon (TOC), iron, bromides, phosphates and color.

The samples were collected and marked in accordance with the obligatory standards and methods [44,45] (Tables S6 and S7).

Samples were collected and analyzed at each stage of the purification process: raw backwash water (RBWW), pre-filter backwash water (PBWW), ultrafiltration backwash water (UBWW), ozonation backwash water (OBWW).

Each backwash water sample was analyzed 3 times, the result of each parameter was averaged from the 3 analyzes performed. Standard deviations of the replicates did not exceed 5%. The presented results (Table S1) are the average values from 5 backwash water treatment cycles.

## 4. Conclusions

The study performed an innovative installation for backwash water treatment. It was found that application of subsequent backwash water treatment processes in the following system: flocculation, pre-filtration, ultrafiltration and ozonation allows them to supply the swimming pool circuit. The designed system for backwash water treatment turned out to be effective in reducing microbiological and physicochemical parameters. During the treatment of microbial contamination, a significant decrease in the values of CFU and Pseudomonas aeruginosa was observed after the use of a pre-filter. However, it was only the use of nanofiltration and ozonation that allowed the obtained values to fall within the requirements for swimming pool water. Ultimately, the effectiveness of removing microbial contamination was achieved at a level of over 99%. Turbidity and oxidability are important physicochemical parameters affecting the quality of swimming pool water. After the application of the system, a decrease in these parameters was found by 99 and 94%, respectively. The backwash water subjected to the purification process meets the stringent requirements of the DIN standard and Polish law relating to swimming pool waters. The use of a filter tube system for rinse water purification is environmentally friendly and the system is an excellent solution for recovering backwash water for swimming pool purposes, allowing up to 96% of the water to be recycled into the circuit. The use of the system enables savings to be achieved in water supply, wastewater management and energy consumption. Thanks to the use of the system, annual savings can reach up to EUR 9000. According to the calculations, the presented system pays off after 2 years of use.

## Figures and Tables

**Table 1:** Cost reduction after using the system with filter tubes.

	Without System	With System	Reduction	Price per Unit, EUR	Annual Savings, EUR
Wastewater discharge, m^3^/y	3989	159	3830	1.2	4596
Drinking water supply, m^3^/y	3989	159	3830	0.8	3064
Energy consumption, kWh/y	88,082	9125	78,957	0.04	3158

**Table 2:** Characteristics of the tube and the operational parameters of the process.

Fibers Material	Cut-Off Molar Mass, kDalton	Filtration Area, m^2^	Specific Flux (Water) at 0.6 atm., Lt/hr/m^2^	Working Pressure, Atm.	Filtration Rate (Absolute), nm
Polysulfone/ Polynephron	67	1.8–2.0	60–80	0.3–0.6	30

## Data Availability

The data presented in this study are available on request from the corresponding author.

## Supplementary Materials

The following are available online, Table S1: Average results obtained during backwash water treatment process, Table S2: Comparison of swimming pool backwash water recovery systems, Table S3: Characteristics of the swimming pool facility, Figure S1: Ultrafiltration installation, Table S4: Microbiological requirements to be met by water in swimming pools, Table S5: Physicochemical requirements to be met by water in swimming pools, Table S6: Summary of the analyzed microbiological parameters and test methods, Table S7: Summary of the analyzed physicochemical parameters and test methods.

## Sample Availability

Samples of the compounds are available from the authors.
