# Supplementary material for: Application of the Swimming Pool Backwash Water Recovery System with the Use of Filter Tubes
Source: Molecules. 2021 Oct 31;26(21):6620. doi: 10.3390/molecules26216620 (PMC8587003; doi:10.3390/molecules26216620)
Supplement: Supplementary file 1 [file molecules-26-06620-s001.zip › molecules-1400793-supplementary.pdf]

Supplementary Materials

Application of the swimming pool backwash water recovery system with the use of filter tubes

Waldemar Studziński[1*] , Wojciech Poćwiarodowski [1,2],Weronika Osińska [2]

[1] Faculty of Chemical Technology and Engineering, Bydgoszcz University of Science and Technology, Seminaryjna 3, 85-326 Bydgoszcz, Poland

[2] Research and Development Center AS PRODUKT, Zajezierze 5c, 88-140 Gniewkowo, Poland

* Corresponding author: Wademar.Studzinski@pbs.edu.pl Faculty of Chemical Technology and Engineering, Bydgoszcz University of Science and Technology, Seminaryjna 3, 85-326 Bydgoszcz, Poland,

Tel.: +48 52 374 90 67, fax.: +48 52 374 90 05

Table S1. Average results obtained during backwash water treatment process

Parameter	Unit	RBWW	PBWW	UBWW	OBWW
Escherichia coli	CFU/100ml	0	0	0	0
Pseudomonas aeruginosa	CFU/100ml	38	20	1	0
**Colony-forming units (CFU) at (36 ± 1)°C**	CFU/ml	16200	800	140	20
**Coagulase-positive staphylococci**	CFU/100ml	0	0	0	0
Legionella sp.	CFU/100ml	0	0	0	0
**Ammonium ion**	mg/l	0.23	0.055	0.059	0.072
**Nitrate concentration**	mg/l	2.1	2.1	1.8	2
**Colour**	mg/l Pt	336	10	2	0.5
**Phosphates**	mg/l	1.06	<0.025	<0.025	<0.025
**Oxidizability**	mg/l	6.26	3.1	1.09	0.4
**Free chlorine**	mg/l	0.05	0.03	0.03	0.02
**Combined chlorine**	mg/l	0.08	0.07	0.04	0.04
**chloroform**	mg/l	0.018	0.013	0.009	0.007
**Turbidity**	NTU	96.9	1.12	0.09	0.13
**Σ THM**	mg/l	0.026	0.018	0.013	0.011
**Ozone**	mg/l	0.01	0.01	0.01	0.03
**Redox potential**	mV	775	755	756	765
**pH**	-	8.4	7.2	7.2	7.3
**Iron**	mg/l	317	<10	<10	<10
**Total Organic Carbon (TOC)**	mg/l	22	5.8	4.3	4.3
**Bromides**	mg/l	0.17	0.16	0.16	0.16

Table S2. Comparison of swimming pool backwash water recovery systems

Comparison of swimming pool backwash water recovery systems					
System name	system with filter tubes	UltraEcoSwim[1]	Lumi-ULTRA[2]	Purified Pool Water System[3]	AS-POOLREC[4]
Country	Poland	Slovenia	Poland	USA	Czech Republic
Prize [thousands €]	30	50-91	65	200	n.d.
Efficiency [m^3/h]	2.5-15	2.5-10	1.5-10	13-51	0.21-10
Electrical power [kW]	5.5	3.00-5.80	1.0-3.5	11.25-30	0.75-5.2
Water recovery [%]	96	75-80	80-95	90	85-97
System components	• wash water reservoir<br>• pre-filter<br>• filter tubes<br>• ozonator<br>• circulating pump<br>• rinsing pump	• ultrafiltration membranes<br>• circulation pump<br>• rinsing pump<br>• treated water tank<br>• pre-filter<br>• chemical dosing pumps<br>• activated carbon filter<br>• carbon filter<br>• circulation pump	• ultrafiltration membranes<br>• circulation pump<br>• rinsing pump<br>• treated water tank<br>• pre-filter<br>• chemical dosing pumps	• chemical dosing pumps<br>• High Pressure Multiple Membrane Filtration Process<br>• circulating pump<br>• rinsing pump	• ultrafiltration membranes or MBR lub RO

n.d.- no data

1- http://ultraecoswim.pl/opis-systemu/
2- https://lumi-tech.pl/oferta/lumi-ultra/
3- https://www.poolwaterpurificationproducts.com/pool-water-purification-products-business
4- https://www.asio.cz/cz/recyklace-bazenovych-vod

Table S3. Characteristics of the swimming pool facility

Information about the swimming pool facility	
Number of filters in the swimming pool system	2
Filter bed diameter	2000 nm
Filter bed area	3.14 m^2
Type of filter bed	Gravel - coal
Filtration velocity	48 $m \cdot h^{-1}$
Rinsing velocity	30 $m \cdot h^{-1}$
Rinsing frequency	Rinsing every 4 days
Type of disinfectant	Sodium chlorate (I)
Temperature in the swimming pool basin	30-32˚C
The amount of water used for rinsing a single filter	10-14m^3
Monthly consumption of tap water to replenish the shortages	420 m^3
Type of coagulant	Floktix DB – aluminum chloride
Number of bathing persons	66 persons per hour<br>205 000 per year

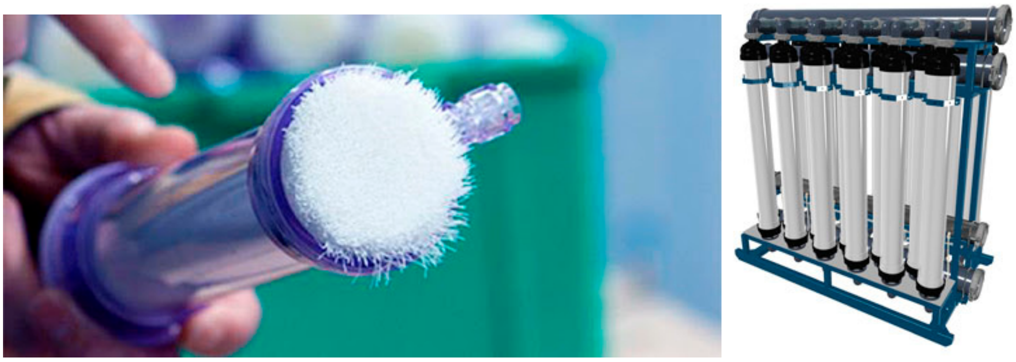

Figure S1. Ultrafiltration installation

Table S4. Microbiological requirements to be met by water in swimming pools

Parameter	Unit	Supply water		Pool water	
		RMH 2015 PL	DIN 19643-1	RMH 2015 PL	DIN 19643-1
Escherichia coli	CFU/100ml	0	0	0	0
Pseudomonas aeruginosa	CFU/100ml	0	0	0	0
**Colony-forming units (CFU) at (36 ± 1) °C**	CFU/ml	20	20	100	100
**Coagulase-positive staphylococci**	CFU/100ml	–	-	–	-
***Legionella* sp.**	CFU/100ml	0	<1	0	<1

Table S5. Physicochemical requirements to be met by water in swimming pools

Parameter	Unit	Supply water				Pool water			
		min		max		min		max	
		RMH 2015 PL	DIN 19643-1	RMH 2015 PL	DIN 19643-1	RMH 2015 PL	DIN 19643-1	RMH 2015 PL	DIN 19643-1
**Turbidity**	NTU	–	–	0.3	0.2	–	-	0.5	0.5
**Chlor wolny**	mg/l	–	0.3	–	0.7	0.3	0.3	0.6	0.6
**Combined chlorine**	mg/l	–	–	0.2	0.2	–	-	0.3	0.2
**Chloroform**	mg/l	–	–	0.03	–	–	–	0.03	0,02
**Σ THM**	mg/l	–	–	0.1	–	–	–	0,1	
**Aluminium**	mg/l	–	–	–	–	–	–	0.2	0.05
**Iron**	mg/l	–	–	–	–	–	–	0.2	0.02
**Nitrate concentration**	mg/l	–	–	20	–	–	–	20	20
**Ozone**	mg/l	–	–	0.05	–	–	–	–	–
**Isocyanuric acid**	mg/l	–	–	–	–	–	–	100	–
**Oxidizability**	mg/l	–	–	–	0.5	–	–	4	0.75
**Redox potential**									
**a) 6,5 ≤ pH ≤ 7,3**	mV	–	–	–	–	750	750	–	–
**b) 7,3 < pH ≤ 7,6**		–	–	–	–	770	770	–	–

RMH 2015 PL - Regulation of the Minister of Health from November 2015 on the requirements that water in swimming pools should meet (Journal of Laws 2015, item 2016) (Poland)
DIN 19643-1- Norm DIN 19643-1:2012-11, Treatment of water of swimming pools and baths. Part 1. General requirements, 2012 (German)

Table S6. Summary of the analyzed microbiological parameters and test methods

Parameter	Unit	Method	Test method
E. coli	CFU/100ml	Membrane filtration method	PN-EN ISO 9308-1:2014-12<br>PN-EN ISO 9308-1:2014-12/A1:2017-04
P. aeruginosa	CFU/100ml	Membrane filtration method	PN-EN ISO 16266:2009
**Coagulase-positive staphylococci**	CFU/100ml	Membrane filtration method	PN-Z-11001-3:2000
***Legionella* sp.**	CFU/100ml	Membrane filtration method	PN-EN ISO 11731-2:2008
**Colony-forming units (CFU) at (36 ± 1) °C**	CFU/ml	Plate method, deep inoculation	PN-EN ISO 6222:2004

Table S7. Summary of the analyzed physicochemical parameters and test methods

Parameter	Unit	Method	Test method	Measuring tools
**Ammonium ion**	mg/l	Photometric	PN-ISO 7150-1:2002	DR 3900 spectrophotometer with RFID technology (Hach, Loveland, CO, USA); Photolyser 400 photometer (Dinotec, Sevilla, Spain)
**Nitrate concentration**	mg/l	Photometric	PN 82/C-04576/08; PN-EN ISO 13395:2001	DR 3900 spectrophotometer with RFID technology (Hach, Loveland, CO, USA); Photolyser 400 photometer (Dinotec, Sevilla, Spain)
**Oxidizability**	mg/l	Titrimetric	PN-EN ISO 8467:2001	Titration kit
**THM (Chloroform)**	mg/l	Gas Chromatography	PN-EN ISO 10301:2002	Chromatograf Agilent Technologies GC7890B with MSD5977A mass detector, USA;
**Σ THM (chloroform bromodichloromethane dibromochloromethane bromoform)**	mg/l	Gas Chromatography	PN-EN ISO 10301:2002	Agilent Technologies GC7890B chromatograph
**Free chlorine**	mg/l	Photometric	PB-25/P wyd. 5 z dnia 20.04.2017; EN ISO 7393-1:2000; PN-EN ISO 7393-2:2018-04	Photolyser 400 photometer
**Combined chlorine**	mg/l	Indirect, calculated		Photolyser 400 photometer
**Ozone**	mg/l	Photometric	Test HACH: LCK 310; Test Dinotec	DR 3900 spectrophotometer with RFID technology
**Turbidity**	NTU	Nephelometric	PN-EN ISO 7027-1:2016-09	Turbidimeter TN-100 (Eutech)
**pH**	-	Potentiometric	PN-EN ISO 10523:2012	SensION meter + MM150 DL (Hach, Loveland, CO, USA)
**Redox potential**	mV	Potentiometric	DIN38404-6	SensION meter + MM150 DL
**Temperature**	°C	Potentiometric	PN-C-04584:1977	SensION meter + MM150 DL
**Total Organic Carbon (TOC)**	mg/l	High temperature combustion with IR detection	PN-EN 1484:1999	NDIR detector
**Iron**	mg/l	Photometric	PB-71 29.10.2019	Photolyser 400 photometer
**Phosphates**	mg/l	Photometric	PN-EN ISO 6878:2006 p.4+Ap1:2010+Ap2:2010	Photolyser 400 photometer
**Colour**	mg/l Pt	Photometric	PN-EN ISO 7887:2012 p.6	Photolyser 400 photometer
